# Case Report: Two Novel *L1CAM* Mutations in Two Unrelated Chinese Families With X-Linked Hydrocephalus

**DOI:** 10.3389/fgene.2022.810853

**Published:** 2022-04-29

**Authors:** Hang Zhou, Qiuxia Yu, Yingsi Li, Fang Fu, Ru Li, Guilan Chen, Dan Wang, Yan Lu, Xin Yang, Dongzhi Li, Can Liao

**Affiliations:** Department of Prenatal Diagnostic Center, Guangzhou Women and Children’s Medical Center, Guangzhou Medical University, Guangzhou, China

**Keywords:** *L1CAM*, hydrocephalus, exome sequence, prenatal diagnosis, case report

## Abstract

L1 cell adhesion molecule is a type I transmembrane glycoprotein belonging to the immunoglobulin superfamily. Pathogenic mutations of *L1CAM* can cause L1 syndrome, referred to as a variety of disease spectrums characterized by hydrocephalus. In the present study, we reported two novel variants of *L1CAM* in two unrelated Chinese families with fetal hydrocephalus history. The woman of family 1, with three consecutive adverse birth histories of male fetuses with hydrocephalus, was identified by an exome sequence with a heterozygous mutation in the *L1CAM* gene, NM_000425.4: c.1696_1703 + 14del (p. S566Vfs*35), which was predicted to be pathogenic. It is predicted to disrupt RNA splicing and likely leads to an absent or disrupted protein product. In family 2, the mother, previously with once a voluntary termination of pregnancy owning to the fetus with hydrocephalus, was pregnant with a fetus with hydrocephalus in her second pregnancy. After fetal blood sampling, a pathogenic deletion of 1511bp in *L1CAM*, chromosome X: 153131395-153132905(hg19/GRCh37)/NM_000425.4: c.2043_2432-121del1511 leading to deletion of fibronectin type-III repeats I-II, was identified in the fetus with hydrocephalus inherited from the mother by an exome sequence. On her third pregnancy, a healthy female fetus was born without the *L1CAM* variant by preimplantation genetic testing for the monogenic disorder. This study emphasizes the importance of ultrasonic manifestation and family history of fetal hydrocephalus for *L1CAM* diagnosis. Our study expands the genotypes of *L1CAM* and aids the genetic counseling of fetal hydrocephalus and even preimplantation genetic testing for the monogenic disorder.

## Introduction

Hydrocephalus is an important birth defect and not rarely seen in fetuses. The incidence of hydrocephalus is approximately 4.65 per 10,000 live births (Garne et al., 2010). X-linked hydrocephalus due to aqueductal stenosis is the most common genetic cause of fetal hydrocephalus (Schrander-Stumpel and Fryns, 1998). Mutations in L1 cell adhesion molecule (L1CAM) were established as a pathogenic factor in X-linked recessive neural disorders (Rosenthal et al., 1992; Adle-Biassette et al., 2013).

L1 cell adhesion molecule (L1CAM) belongs to the immunoglobulin supergene family and plays an essential role in neuronal migration, neuronal cell survival, development, and differentiation (Kenwrick et al., 2000). L1 syndrome is defined as a variety of phenotypes caused by mutations of *L1CAM*, including CRASH syndrome (corpus callosum hypoplasia, retardation, adducted thumbs, spastic paraplegia, and hydrocephalus; OMIM 303350), hydrocephalus due to stenosis of the aqueduct of Sylvius (HSAS; OMIM 307000), and hydrocephalus with Hirschsprung disease (OMIM 307000) (Vos et al., 2010).

To date, 292 pathogenic mutations of L1CAM were identified according to the Human Gene Mutation Database (HGMD). The characteristics of mutations revealed 36.8% missense mutations, 18.6% splice site changes, and 22.3% deletions. The classical phenotypes induced by mutations in *L1CAM* mainly included hydrocephalus, accompanied by other disorders. Here, we described two novel variants of *L1CAM*, which were both unreported previously, including NM_000425.4:c.1696_1703 + 14del (p.S566Vfs*35) and the intragenic base deletion of 1511bp, chromosome X: 153131395-153132905(hg19/GRCh37)/NM_000425.4: c.2043_2432-121del1511, in two unrelated families from the Chinese mainland.

## Case Presentation

### Case 1

**FIGURE 1:**
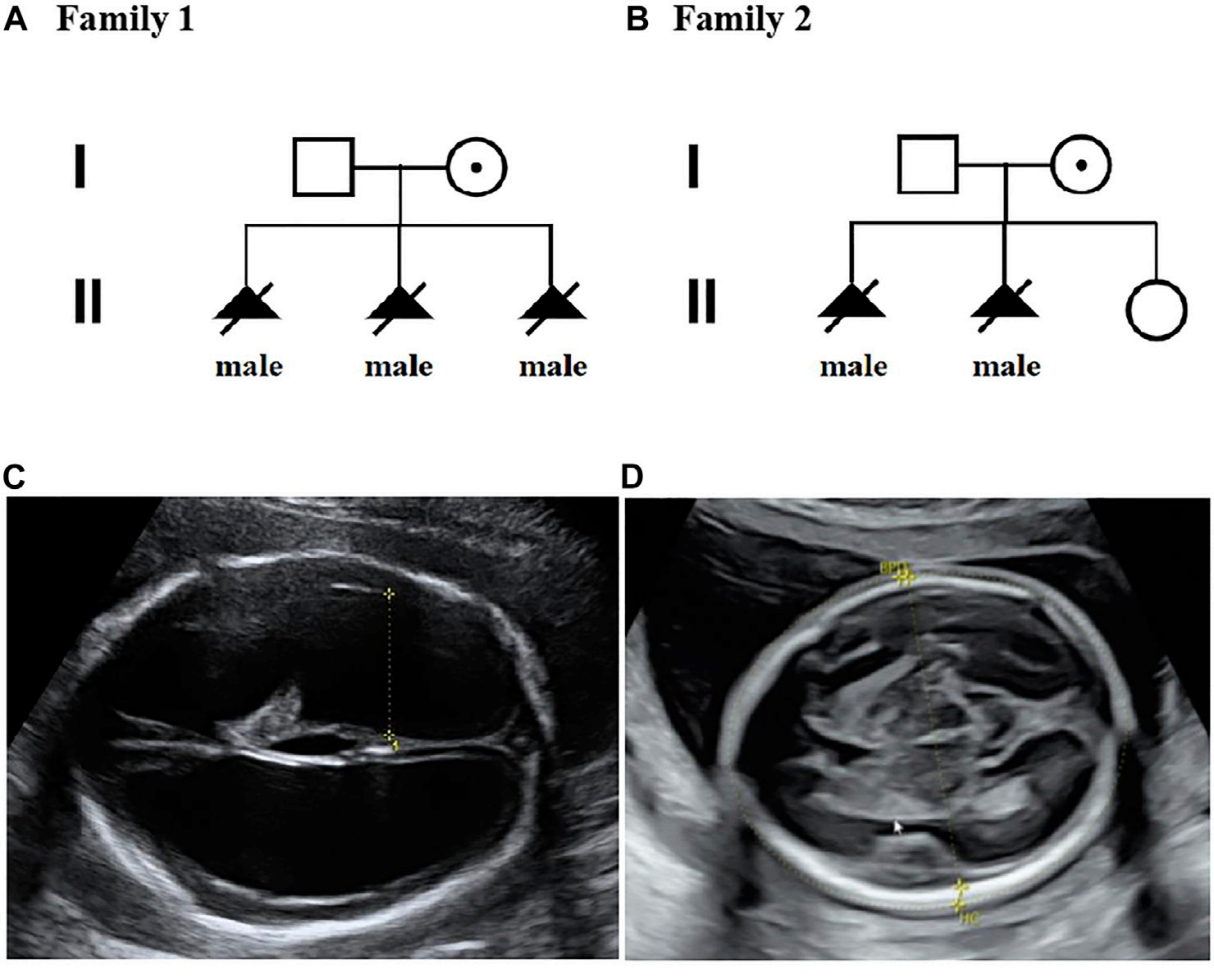
Pedigrees of two families with X-linked hydrocephalus; **(A)** Pedigree of family 1; **(B)** Pedigree of family 2; **(C)** Ultrasound view of the second fetus with severe hydrocephaly from family 2; **(D)** No hydrocephalus was found in II-3 individual with normal anomaly scan of ultrasound from family 2.

**FIGURE 2:**
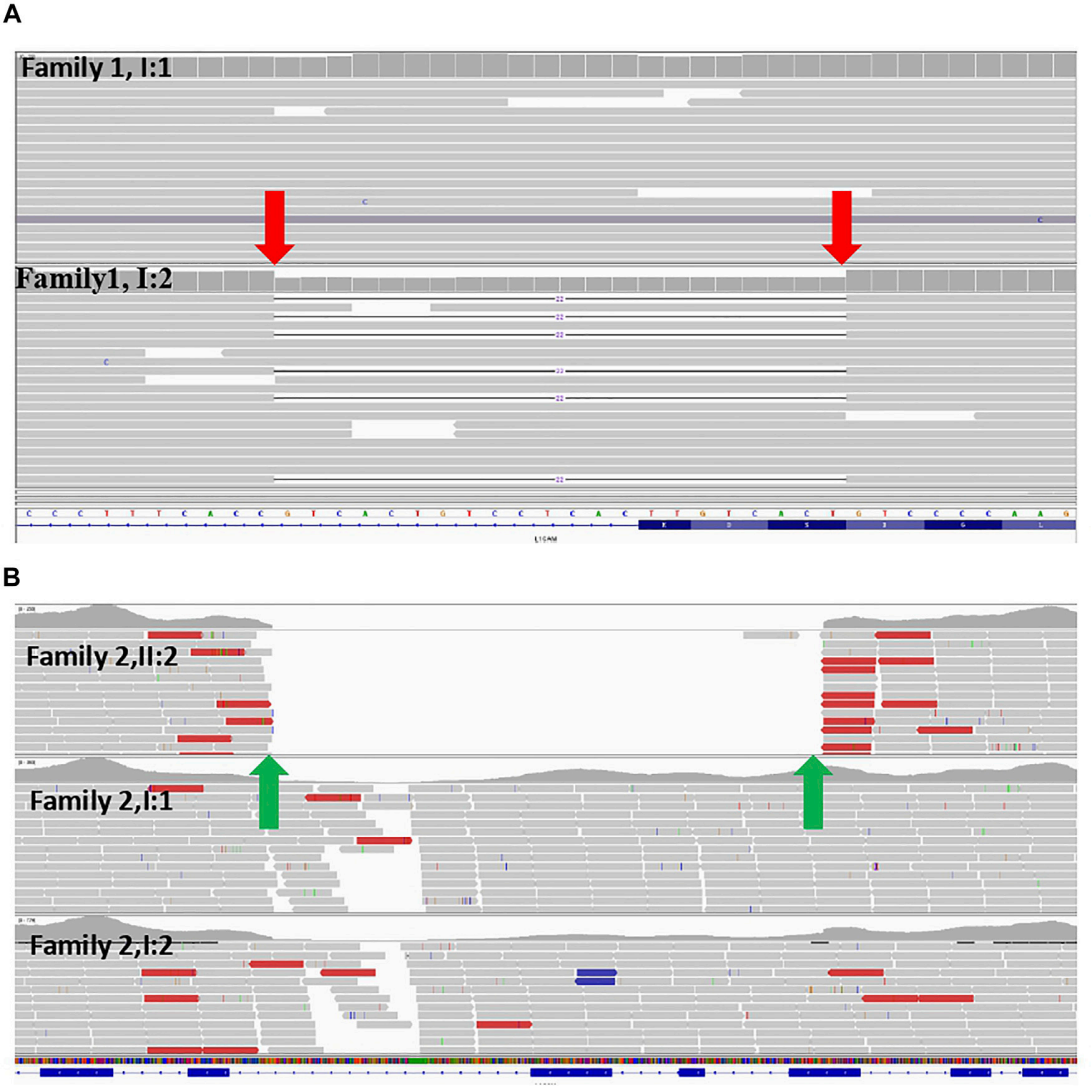
**(A)** Exome sequence for the couple from family 1 indicated that the woman was a carrier of the variant (NM_000425: c.1696_1703 + 14del) in the *L1CAM* gene (red arrows) **(B)** Exome sequence for family 2 displayed that the deletion of 1511bp in *L1CAM* chromosome X: 153131395 (hg19/GRCh37)/NM_000425.4:c.2043_2432-121del1511 in the II-2 fetus (green arrows).

**FIGURE 3:**
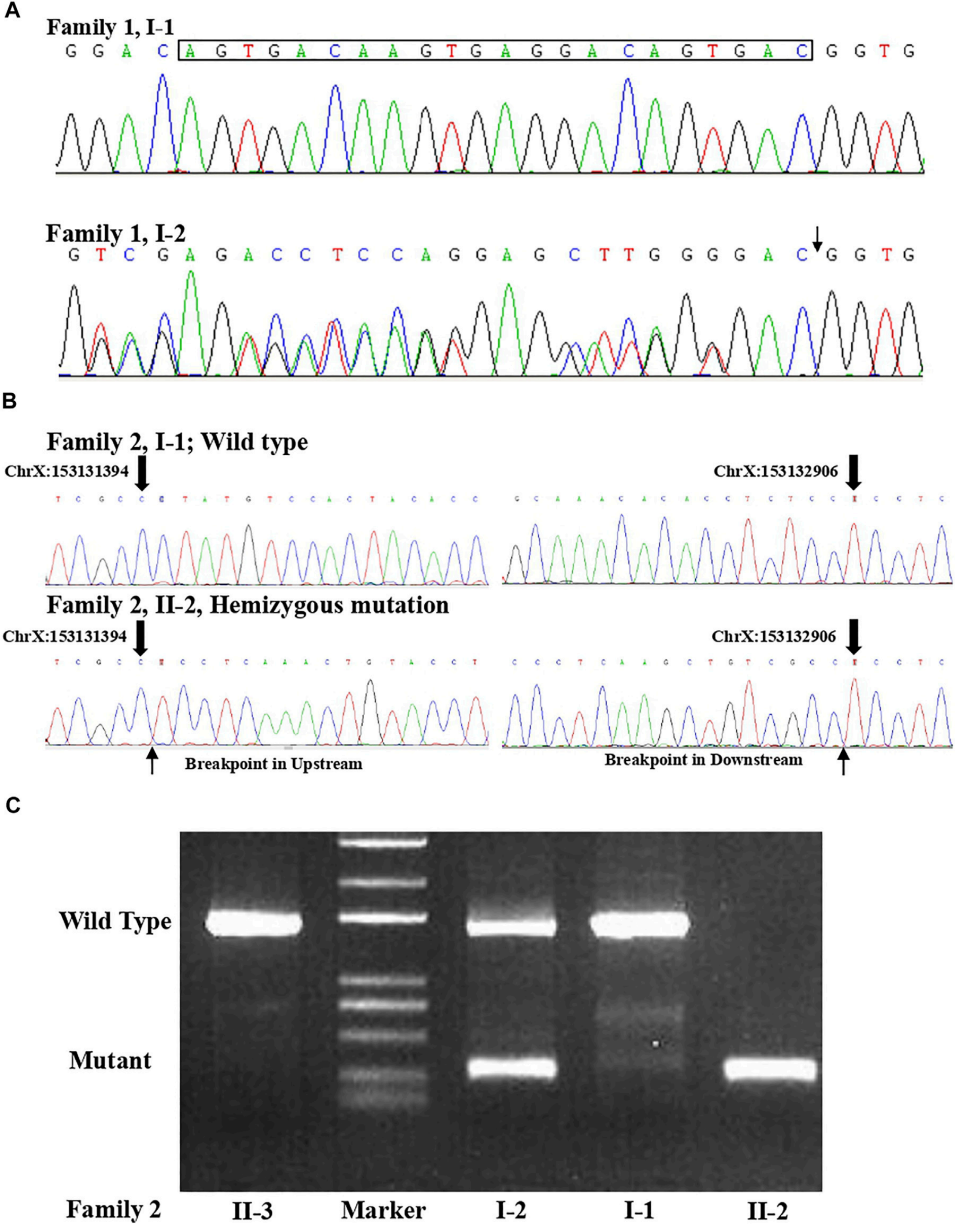
**(A)** Sanger sequence for the couple from family 1 confirmed that the woman was a carrier of the variant (c.1696_1703 + 14del) in the *L1CAM* gene. **(B)** Sanger sequence result for family 2 validated the deletion (c.2043_2432-121del) in *L1CAM* in II-2 fetus. **(C)** Agarose gel electrophoresis in the *L1CAM* result from family 2.

A non-pregnant woman came to our center of prenatal diagnosis for consultation after three consecutive adverse birth histories of fetuses with hydrocephalus but without image data. All fetuses were male. The pedigree is shown in Figure 1A. However, there were no chromosomal or genetic tests for the three fetuses. Karyotype results in this couple did not reveal any abnormality. The couple wanted to find out the etiology of the three consecutive adverse birth histories of hydrocephalus. We offered a whole-exome sequence (Figure 2A) for this couple and found a novel pathogenic heterozygous variation in the *L1CAM* gene in the woman, NM_000425.4: c.1696_1703 + 14delAGTGACAAGTGAGGACAGTGAC(p.S566Vfs*35). Sanger sequence results for the couple confirmed this variant (Figure 3A). But the variant has yet to be reported or present in population databases (1000 Genome Project, ExAC, EVS, and gnomAD). This variant is a deletion of part of exon 13 (c.1696_1703 + 14del), which may disrupt functions of the Ig-like C2-type 6 domain in the *L1CAM* gene. It is predicted to disrupt RNA splicing and likely leads to an absent or disrupted protein product. It is highly associated with the *L1CAM* gene and the phenotype of three consecutive males with hydrocephalus. Based on the standards and guidelines of the American College of Medical Genetics and Genomics, the variant was predicted to be pathogenic (PVS1+PM2_Supporting + PP4). However, her subsequent fertility condition cannot be acquired by a telephone follow-up.

### Case 2

A 36-year-old woman was in her second pregnancy at 24 weeks of gestation (Figure 1B). She was referred to our prenatal diagnosis department because of fetuses diagnosed with hydrocephalus and invisible cavum septum pellucidum by ultrasound. We confirmed hydrocephalus but did not find invisible cavum septum pellucidum by ultrasound (Figure 1C). In the first trimester, there was a normal ultrasound examination, and the nuchal translucency measurement was 1.5 mm. Nevertheless, she previously had one voluntary termination of pregnancy owning to the fetus with hydrocephalus in 24 gestational weeks, but genetic tests were not performed for this fetus. This couple was both healthy and not consanguineous. There were no abnormal karyotype results in the couple. After informed consent, we performed cordocentesis on this woman. The TORCH-IgM and karyotype results were normal. But trio medical exome sequencing identified a copy number variation in *L1CAM* inherited from his mother in this male fetus, chromosome X: 153131395-153132905(hg19/GRCh37)/NM_000425.4: c.2043_2432-121del1511 (Figure 2B); this structural variant was confirmed by Sanger sequence (Figure 3B), whereas it has not been previously reported, and it was predicted to cause disruption in partial exon 16 and whole-exons 17 and 18, leading to deletion of fibronectin type-III repeats I-II. The parents requested to terminate the pregnancy but refused autopsy at 25 gestational weeks. After that, she chose preimplantation genetic testing for monogenic disorders (PGT-M) to gain an embryo with an absence of this mutation in her third pregnancy. Amniocentesis was performed in our department at 17 weeks of pregnancy, showing and confirming the absence of this mutation (Figure 3C; II-3). Chromosomal microarray analysis was normal. This healthy female fetus showed a normal anomaly scan of ultrasound in the second trimester (Figure 1D) and was born at 38 weeks of gestation by cesarean section for social reasons. Her birth weight was 3.0 kg, and her head circumference was 33.1 cm. Apgar scores were 9 and 10 at 1 and 5 min after birth, respectively. There was no report of hydrocephalus and mental disorders through the telephone follow-up.

## Material and Methods

### Karyotype and Chromosomal Microarray Analysis

To determine the karyotype of fetal cord blood, amniocytes, and peripheral blood, colchicines were used to arrest samples at the metaphase by conventional karyotyping. G-banding karyotype was performed at the 320–400 band level by analyzing 20 split phases. CMA analysis was performed using the Affymetrix CytoScan HD GeneChip Platform (Affymetrix, Santa Clara, CA, United States) with copy number variation (CNV) and single nucleotide polymorphism (SNP) probes, following the manufacturer’s instructions. Genomic DNA was isolated from samples by using a QIAamp DNA Blood Midi/Mini kit (QIAGEN GmbH; Hilden, Germany). The CNVs were reported at a 100-kb threshold with a marker count of ≥50.

### Exome Sequence

Target enrichment in DNA samples was performed using Agilent SureSelect human exome capture probes (V6, Life Technologies, United States), according to the manufacturer’s protocol. The DNA library was sequenced on Hiseq XTen (Illumina, Inc., San Diego, CA, United States) for pair-end 150-bp reads. Raw reads were filtered using Trimmomatic to remove adapter-contaminated reads and low-quality reads. Clean reads were aligned to the human reference genome (hg19/GRCh37) with BWA. BAM files were generated by SNP analysis, duplication marking, indel realignment, and recalibration by GATK and SAMtools. The minor allele frequencies (MAFs) of all known variants were annotated according to dbSNP, the 1000 Genome Project, ExAC, EVS, gnomAD, and our in-house database. Databases such as OMIM, ClinVar, and the Human Gene Mutation Database were used to determine mutation harmfulness and pathogenicity where appropriate. To predict biological effect analysis of candidate variant genes, multiple computational algorithms were used, including SIFT, MutationTaster, PolyPhen2, PROVEAN, CADD, Human Splicing Finder, MaxEntScan, and NNSplice.

## Discussion

L1 syndrome is referred to as an X-linked recessive disorder and includes broad phenotypic spectrums dominated by hydrocephalus. The diagnostic yield can become higher when identifying characteristics of the L1 syndrome and family history (Adle-Biassette et al., 2013). The detection rate of the L1 syndrome can reach up to 32% for patients with at least two additional cases (Ochando et al., 2016). Therefore, the clinician should not overlook this syndrome when hydrocephalus in fetuses is recognized by prenatal ultrasound, especially in women with an abnormal reproductive history of a male fetus.

From the mutation characteristics in *L1CAM*, variants of L1CAM seem unique in families, and there is no hotspot mutation. However, recent studies revealed the correlations between genotypes and phenotypes in the *L1CAM* gene. Phenotypic severity primarily depends on the location and type of mutation of *L1CAM* (Fransen et al., 1998). Extracellular mutations resulting in truncation or absence of *L1CAM* lead to a more severe phenotype. Milder to severe phenotype could be caused by missense mutations in the extracellular part. Cytoplasmic mutations tend to generate a milder phenotype which might affect signal transduction or interaction with the cytoskeleton (Sebens Müerköster et al., 2007).

The woman from family 1 carried a novel heterozygous variant NM_000425.4: c.1696_1703 + 14del (p.S566Vfs*35). So we strongly believed that all the three consecutive male fetuses with hydrocephalus were with the hemizygous pathogenic mutation, although samples were unavailable. This variant results in a deletion of part of exon 13 and is predicted to disrupt RNA splicing and likely leads to an absent or disrupted protein product. It might lead to a more severe phenotype from the report of genotype–phenotype correlation in *L1CAM* (Fransen et al., 1998). Although this variant has not been reported in the literature with *L1CAM*-related diseases, it is known that the variant with loss-of-function in *L1CAM* was pathogenic (Vos et al., 2010). A similar variant NM_000425.5: c.1702_1703 + 14del reported from ClinVar (rs1603275315) was classified as likely pathogenic, showing spastic paraplegia. Furthermore, this variant from family 1 affected the integrity of the sixth Ig domain of *L1CAM*. The Ig-like domains of L1CAM play a major role in interactions with several extracellular ligands such as phosphacan and neurocan, integrins, axonin-1/TAG1, Sema 3A, and also itself (Ruppert et al., 1995; Castellani et al., 2000). Another report has also shown that an individual with a variant of splice site change (c. 1704-1G > A) was diagnosed with adducted thumbs, hydrocephalus, and spastic paraplegia, accompanying abnormal karyotype (47, XXY) (Kanemura et al., 2006). A mutation involving the sixth Ig domain of L1CAM (L1-6D) (c.1759 G > C; p. G587R) was previously associated with developmental delay, dysmorphic facies, adducted thumbs, truncated corpus callosum, and periventricular heterotopias associated with polymicrogyria in a 2.5-year-old boy (Shieh et al., 2015). Investigations of the L1-6D knocked-out mice revealed typical hydrocephalus. The L1-6D isoform lost its ability to bind to *L1CAM* in a homophilic manner and α5β1 integrin (Hortsch, 1996). In another report on L1-6D mice, the phenotype exhibited abnormal ensheathment of unmyelinated axons by Schwann cell processes, eventually leading to myelinated multiaxon bundles (Itoh et al., 2005). In conclusion, currently available evidence displays the pathogenicity of this variant, but further study is needed to confirm the observation.

In family 2, we identified a novel large deletion in *L1CAM* in the second fetus and the pregnant woman. First, there are various types of variants in *L1CAM*, which reminds us that besides employing sequencing to detect point mutation, we should consider using proper approaches to identify different variants in *L1CAM*, when one X-linked hydrocephalus is suspected. Second, there were a few reports of a large deletion in *L1CAM*. To our knowledge, variants of more than 1 kb of deletion were found in our current study, and in addition, three cases of an *L1CAM* whole-gene deletion were previously described in other reports. Knops et al. have reported a 61 kb in size encompassing all of L1CAM, AVPR2, and part of ARHGAP4, leading to L1 syndrome and nephrogenic diabetes insipidus (Knops et al., 2008). This individual was diagnosed to require shunting due to significant hydrocephalus. The second case, identified with an *L1CAM* whole-gene deletion and *PDZD4* gene, was also found with hydrocephalus that needed shunting (Kutsche et al., 2002). Similar to the second case, the third report demonstrated a 4-month-old male with a *de novo L1CAM* whole-gene deletion and *PDZD4* gene (Chidsey et al., 2014). Simultaneous deletions in upstream or downstream were present in all three reports of *L1CAM* whole-gene deletion. Moreover, the variant from family 2 was predicted to cause deletion of fibronectin type-III repeats 1-2 (FN III 1-2). De Angelis et al. demonstrated that mutations located throughout Ig1-Ig6 and Fn2 disrupted both types of interactions by synthesizing L1CAM-Fc fusion proteins of disease-causing variants (De Angelis et al., 1999; De Angelis et al., 2002). One study found that FN III 1-5 in *L1CAM* could interact with the fibroblast growth factor receptor (FGFR), which was essential in neuronal differentiation and FGFR1 phosphorylation. Authors speculated that FN III 2 amino acids (AVNNQGKG) were crucial binding sites (Kulahin et al., 2008). Furthermore, FN III 2 plays an important part in interactions with *L1CAM* and enhances structural stability in the extracellular region (Kunz et al., 1998). Therefore, the variant of family 2 could disturb the interaction with FGFR to cause neural axon malformation, resulting in severe hydrocephalus. Further functional studies of the variant were being conducted in our laboratory.

Preimplantation genetic testing for monogenic disease (PGT-M) is a useful tool for the *in vitro* fertilization (IVF) process (Masset et al., 2019). A biopsy is performed for single or several cells from IVF embryos to exclude the curated disease-causing cells, selectively transferring the unaffected embryos. After the pathogenic variant in *L1CAM* was identified, the couple from family 2 benefited from the PGT technology and eventually delivered a healthy infant without this mutation.

In conclusion, this study described two novel pathogenic variants and phenotypes in two Chinese families, which effectively blocked the transmission of genetic birth defects. These patients presented here may provide insights to better understand the L1 syndrome and probably help genetic counseling and PGT for prenatal X-linked hydrocephalus.

## Data Availability

The original contributions presented in the study are included in the article/Supplementary Material, further inquiries can be directed to the corresponding author.
